# Pumilio-1 mediated translational control of claudin-5 at the blood-brain barrier

**DOI:** 10.1186/s12987-024-00553-5

**Published:** 2024-06-19

**Authors:** Yosuke Hashimoto, Chris Greene, Nicole Hanley, Natalie Hudson, David Henshall, Kieron J. Sweeney, Donncha F. O’Brien, Matthew Campbell

**Affiliations:** 1 Smurfit Institute of Genetics, Trinity College Dublin, Dublin 2, Ireland; 2 Science Foundation Ireland Research Centre for Chronic and Rare Neurological Diseases, FutureNeuro, Royal College of Surgeons in Ireland (RCSI), University of Medicine and Health Sciences, Dublin, Ireland; 3 Department of Physiology and Medical Physics, RCSI University of Medicine and Health Sciences, Dublin, Ireland; 4 Department of Neurosurgery, Beaumont Hospital, Dublin, Ireland

**Keywords:** Claudin-5, Pumilio, Tight junction, Translational regulation

## Abstract

**Abstract:**

Claudin-5 is one of the most essential tight junction proteins at the blood-brain barrier. A single nucleotide polymorphism rs10314 is located in the 3’-untranslated region of claudin-5 and has been shown to be a risk factor for schizophrenia. Here, we show that the pumilio RNA-binding protein, pumilio-1, is responsible for rs10314-mediated claudin-5 regulation. The RNA sequence surrounding rs10314 is highly homologous to the canonical pumilio-binding sequence and claudin-5 mRNA with rs10314 produces 25% less protein due to its inability to bind to pumilio-1. Pumilio-1 formed cytosolic granules under stress conditions and claudin-5 mRNA appeared to preferentially accumulate in these granules. Added to this, we observed granular pumilio-1 in endothelial cells in human brain tissues from patients with psychiatric disorders or epilepsy with increased/accumulated claudin-5 mRNA levels, suggesting translational claudin-5 suppression may occur in a brain-region specific manner. These findings identify a key regulator of claudin-5 translational processing and how its dysregulation may be associated with neurological and neuropsychiatric disorders.

**Graphical Abstract:**

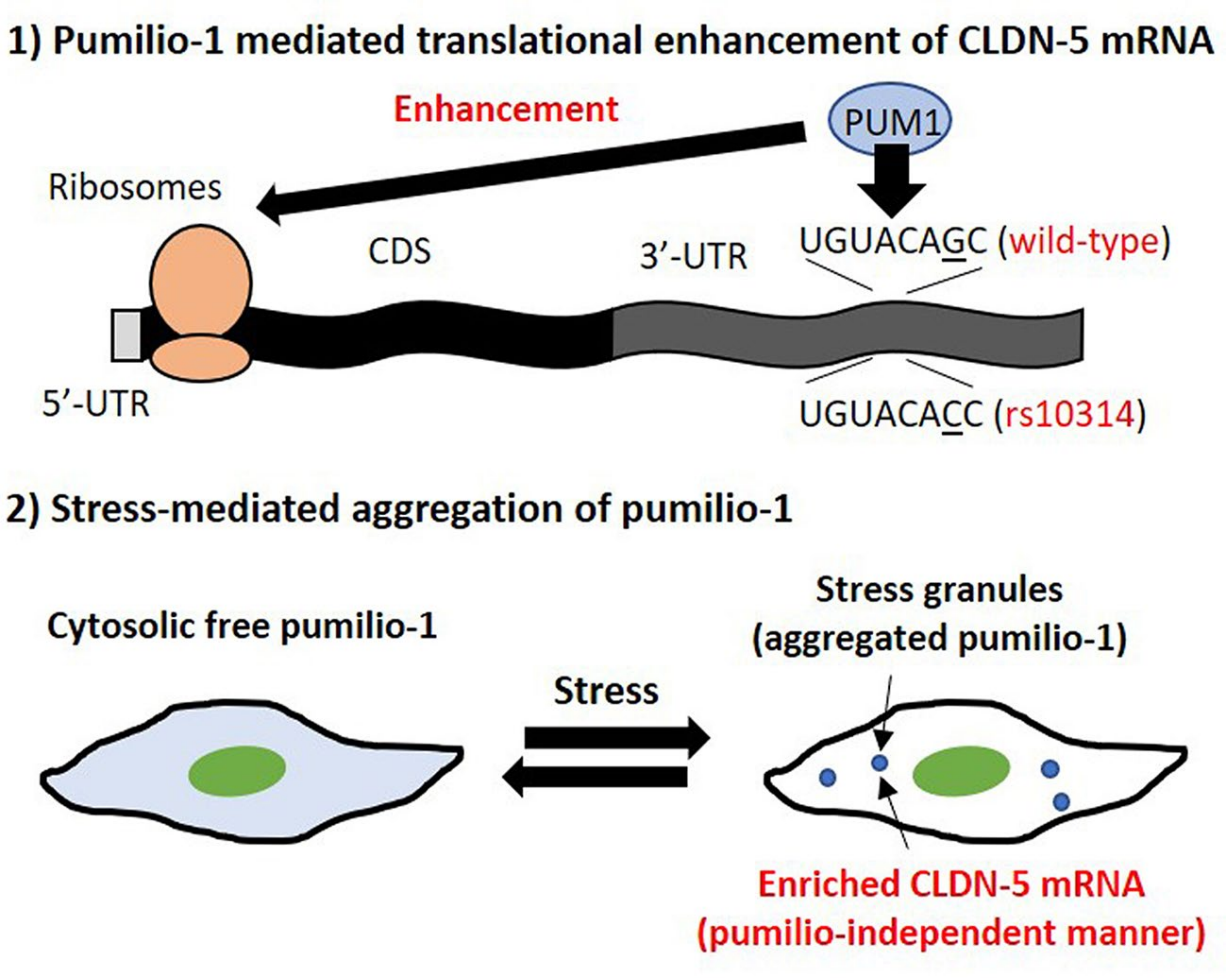

**Supplementary Information:**

The online version contains supplementary material available at 10.1186/s12987-024-00553-5.

## Introduction

Endothelial cells lining the vasculature function as a physical barrier to separate fluid compartments within the body and to enable optimum organ function. In the vasculature associated with the brain, the endothelial barrier properties are stronger than that of peripheral endothelial cells due to their well-developed tight junctions. The limited paracellular diffusion across brain endothelial cell layer allows for the specific uptake of molecules required to maintain the brain microenvironment. Claudins (CLDNs) are four-pass transmembrane proteins that are major components of the tight junctions. CLDNs have 27 identified family members in mammals and many CLDNs are expressed in a tissue-specific manner. Among CLDN family members, CLDN-5 is predominantly expressed in the brain endothelial cells [10, 50] and is considered the major protein that prevents diffusion of molecules less than 1 kDa into the brain parenchyma [6, 37].

Down-regulation or functional inhibition of CLDN-5 has been shown to lead to neurological dysfunction. Mice with sustained inhibition of endothelial CLDN-5 expression by RNA interference developed severe neuroinflammation and seizures and died after 3–4 weeks of the initiation of knockdown [18]. Experiments examining brain region-specific CLDN-5 knockdown showed that increased vascular permeability in different brain regions induced differential behavioural changes in mice [11, 18, 34]. Added to this, non-human primates treated with a monoclonal antibody against the extracellular domain of CLDN-5 suffered epileptic convulsions [46]. In humans, significant DNA methylation in the *CLDN5* locus, located at chromosomal region 22q11, were observed in patients with early stages of Alzheimer disease-associated cognitive decline [25] and patients with heterozygous pathogenic *CLDN5* missense mutants have been reported to present with epilepsy with microcephaly [9, 24]. Additionally, 22q11 deletion syndrome (22q11DS) is known to occur in 1:2,500—1:4,000 live births and 30% of patients with this microdeletion develop schizophrenia [1]. Furthermore, rs10314, a single nucleotide polymorphism (SNP) in *CLDN5*, is known as a weak risk factor of schizophrenia [18, 39][45, 51]. Patients with both 22q11DS and rs10314, whose allele frequency is 16%, showed higher prevalence of schizophrenia [18]. Therefore, indicators of CLDN-5 expression level and permeability of the brain vasculature can be postulated as a potent marker to predict the risk and severity of central nervous system (CNS) diseases [27]. Many molecules/factors are identified as transcriptional enhancers/repressors of *CLDN5* [23] however, translational regulation of *CLDN5* mRNA is very poorly understood and the mechanisms underlying its regulation are not clear.

Previously, we revealed that rs10314 attenuated CLDN-5 protein expression level without changing *CLDN5* mRNA level but with changing polyribosomal profiles [18]. The rs10314 (G to C mutation) is located in a relatively AU-rich region in the 3’-untranslated region (3’-UTR) of *CLDN5* mRNA (Supplementary Fig. S1a). In this regard, many disease-associated SNPs located in the 3’-UTR create or disrupt micro RNA (miRNA)-binding sites [19]. However, we previously reported that the rs10314-mediated down-regulation was not mediated by miRNA [18]. Here, we hypothesized that rs10314 creates or disrupts a binding-site of an unknown RNA-binding protein that controls the translational activity of CLDN-5 mRNA.

Therefore, we performed RNA‒protein pull-down assays to identify the candidate RNA-binding proteins that are responsible for rs10314-mediated translational suppression of CLDN-5 mRNA and identified that the rs10314 mutation attenuated the interaction of pumilio proteins that bind to specific single-stranded RNA sequences. Pumilios (PUMs) function as translational enhancers of CLDN-5 mRNA under physiological conditions. PUMs are a major component of stress granules which are cytosolic membrane-less ribonucleoprotein granules induced by multiple cellular stresses, including oxidative stress (induced by pollutants such as inorganic arsenic), UV irradiation and virus infection [12, 28]. Stress granules protect mRNAs from stress-induced degradation and trapped mRNAs can be released with a translationally active status when cells recover from stress. Stress granule formation is an adaptation process in response to cellular stresses, however, sustained chronic stress induced by pathogenic factors in CNS disease are known to prevent disassembly of stress granules [49, 53]. Intriguingly, we showed that CLDN-5 mRNA preferentially accumulated in stress granules and cytosolically distributed PUM1 dramatically decreased during stress granule formation. Added to this, vascular-localized granular PUM1 was observed in the hippocampus of patients with psychiatric disorders and epilepsy. Taken together, we conclude that PUM1 is a potent translational regulator of CLDN-5 that is abolished by the rs10314 SNP. We also show that PUM1-mediated CLDN-5 regulation is a key mediator of aberrant CLDN-5 expression during cell stress and may represent a novel therapeutic angle for preserving BBB integrity.

## Results

### Identification of pumilio as a binding partner of CLDN5 mRNA

Previously, we discovered that rs10314-mediated CLDN-5 regulation was translational regulation as this mutation did not change the mRNA level but changed polyribosome profiling [18]. To examine the detailed mechanism of rs10314-mediated CLDN-5 regulation, we focused on RNA-binding proteins and performed an RNA–protein pull-down assay followed by proteomic screening. Biotinylated RNAs with the coding sequence (CDS) and 3’-UTR of wild-type or rs10314-type *CLDN5* with an artificial polyA tail (approximately 1250 bases in total, Supplementary Fig. S1a) were transcribed using a T7 promoter and were conjugated with microbeads. The RNA conjugated beads were treated with the lysate of HEK-293 cells and isolated microbeads were subjected to proteomic analysis. Approximately 970 proteins were detected and 127 of those were RNA-binding proteins (Supplementary Table S1). Using a difference in label-free quantification (LFQ) intensity of 1.8-fold or greater as a cutoff threshold, 5 RNA-binding proteins showed differential binding affinity between wild-type and rs10314-type *CLDN5* RNA (Table 1). Two of them preferentially interacted with the wild-type sequence and the others preferentially interacted with the rs10314 sequence. Among them, the binding sequence of Smaug and Pumilio proteins have been reported; Smaug binding element is a stem-loop containing a pentaloop with a CNGGN sequence [38] and Pumilio binding element (PBE) is a single-stranded UGUANAUW (where N is A, C, G or U and W is A or T) sequence. The 3’-UTR of *CLDN5* mRNA can be formed by a pentaloop with CUGGU especially with the help of randomly inserted biotins but this structure is not stable as has been predicted by mFold software [57] (Supplementary Fig. S1b and S1c). We therefore excluded Smaug 2 for further analysis. Additionally, activating signal co-integrator complex (ASCC3) has been reported to interact with stalled ribosomes on mRNAs to relocate them for proper translation [44]. It may be consistent with our previous result that the rs10314 mutation changed polyribosome profiling [18]. The role of Poly(rC)-binding protein 1 (PCBP1) and U5 small nuclear ribonucleoprotein 200 kDa helicase (SNRNP200) for translation is unknown.

**Table 1:** The summary of identified RNA-binding proteins in RNA-protein pull-down products

Protein names	Gene names	LFQ intensity (×1,000)		Ratio
		Wild-type	rs10314	
Pumilio homolog 1	PUM1	78,250	39,809	1.97
Protein Smaug homolog 2	SAMD4B	187,950	73,973	2.54
Activating signal cointegrator 1 complex subunit 3	ASCC3	97,631	274,140	0.36
Poly(rC)-binding protein 1	PCBP1	200,370	387,210	0.52
U5 small nuclear ribonucleoprotein 200 kDa helicase	SNRNP200	618,720	1,148,400	0.54

The label-free quantification (LFQ) intensities of identified RNA-binding proteins, that show more than 1.8-fold difference between wild-type and rs10314 are shown. For the data list of nano-LC-ESI-MS/MS, see Supplementary Table S1

**Fig. 1:**
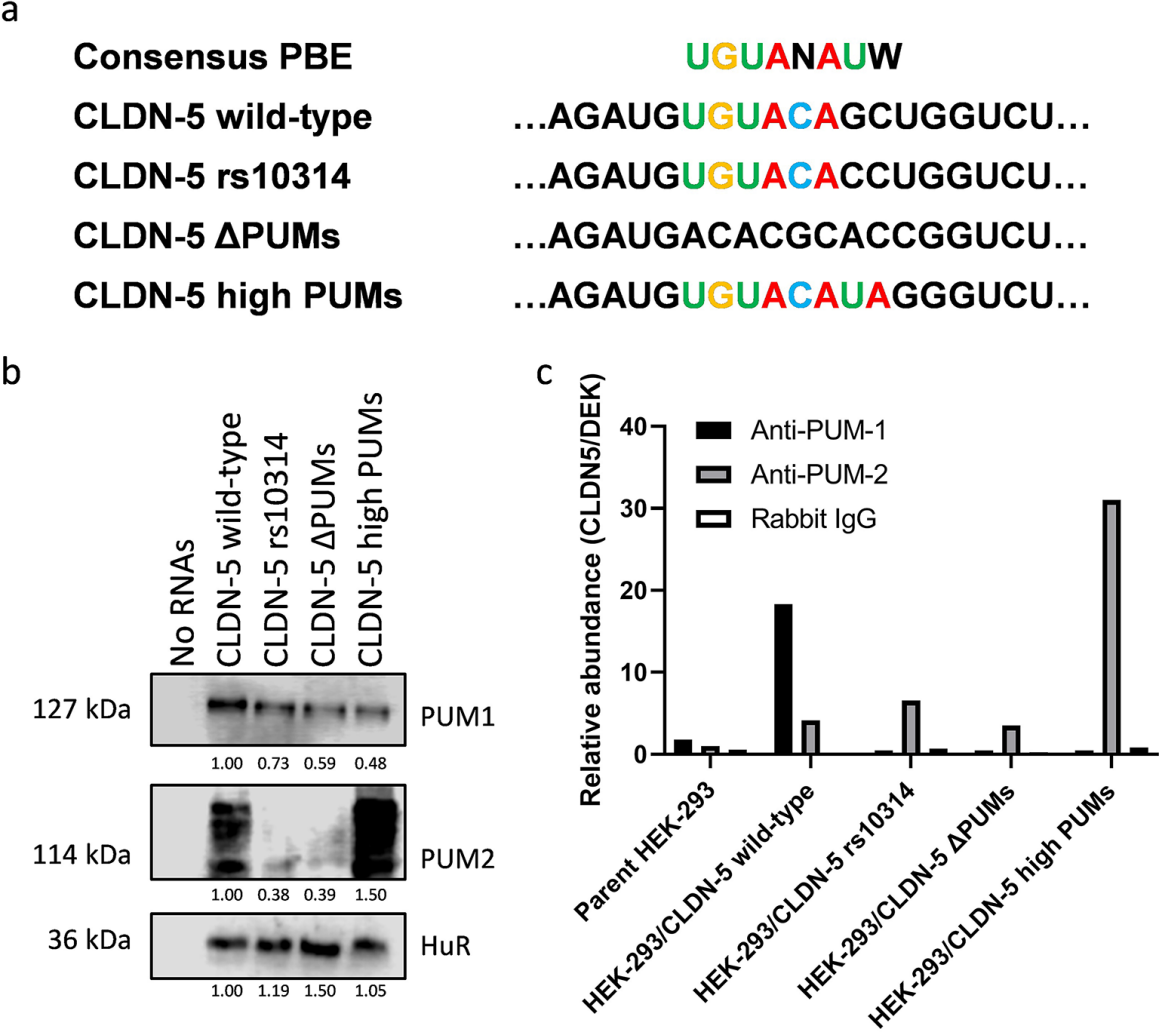
Identification of pumilio proteins as binding partners of CLDN-5 mRNA. **(a)** Sequence alignment of consensus pumilio binding element (PBE) and wild-type and rs10314 CLDN-5. N is A/U/C/G and W is A/T. Two additional 3’-UTR mutants that completely lack pumilio binding ability (ΔPUMs) or have canonical PBE (high PUMs) are also prepared for further studies. **(b)** The presence of pumilios in RNA-protein pull-down products was confirmed by Western blotting. The biotinylated CLDN-5 RNAs prepared from vectors coding wild-type CLDN-5 or its mutant (rs10314, ΔPUMs or high PUMs) were conjugated with microbeads and mixed with the lysate of parent HEK-293 cells. The precipitated magnetic beads were subjected to Western blotting. The abundance of HuR was used as a loading control. **(c)** RNA immunoprecipitation assay. Abundance of CLDN-5 mRNA was normalized by another PUM targeting gene, DEK

Human Pumilio (PUM) 1 and 2 share 69% identity and bind to the same consensus binding sequence; however, their binding preference is slightly different due to the secondary structure of mRNAs [43]. The sequence around rs10314 (UGUACAGC, G at position 7 is C in rs10314) is PBE with two substitutions in the last two bases. Of note, 6 bases and 4 bases of the putative PUM-binding sequence are predicted to be a single-strand in wild-type and rs10314-type sequence, respectively, in the most stable secondary structure of 3’-UTR (Supplementary Fig. S1c). To confirm its binding to PUMs, an RNA–protein pull-down assay was performed with two control sequences whose sequence completely lacked any binding affinity for PUMs (ΔPUMs) or canonical PBE (high PUMs) (Fig. 1a). HuR (ELAVL1) was chosen as a loading control because HuR was detected by proteomic analysis in both samples (Table 1) and the CLDN-5 3’-UTR has an obvious HuR-binding sequence (UUUCCUUUU) [31] (Supplementary Fig. S1a). PUM1 was detected in all 4 pulled-down products including ΔPUMs mutated RNA. However, wild-type CLDN-5 RNA could pull-down a significantly higher amount of PUM1 (Fig. 1b). Wild-type CLDN-5 RNA could also pull-down an appreciable level of PUM2, albeit to a lesser degree than the high PUMs mutated RNA. Intensive post-translational modifications to PUM2 were observed in these pulled-down products. To check whether interaction between PUMs and CLDN-5 mRNA can occur in cells, an RNA-immunoprecipitation (RIP) assay was performed using HEK-293 cells-expressing CLDN-5 with the wild-type 3’-UTR sequence or its mutated sequences. Anti-PUM1 antibodies efficiently isolated only the wild-type CLDN-5 mRNA, while anti-PUM2 antibodies efficiently isolated high PUMs CLDN-5 mRNA (Fig. 1c), indicating that PUMs can be accessible around the rs10314 site. Although it was less efficient, anti-PUM2 antibodies also precipitated all CLDN-5 mRNAs. In contrast to the result of the RNA-protein pull-down assay, PUM2 binding to wild-type CLDN-5 mRNA was not strong. This is likely due the difference in secondary structure of biotinylated RNAs and mRNA. Taken together, these results clearly suggest that PUMs are a potent binding partner of CLDN-5 mRNA and its binding is affected by the rs10314 mutation.

### PUM-binding is essential for translational up-regulation of CLDN-5

**Fig. 2:**
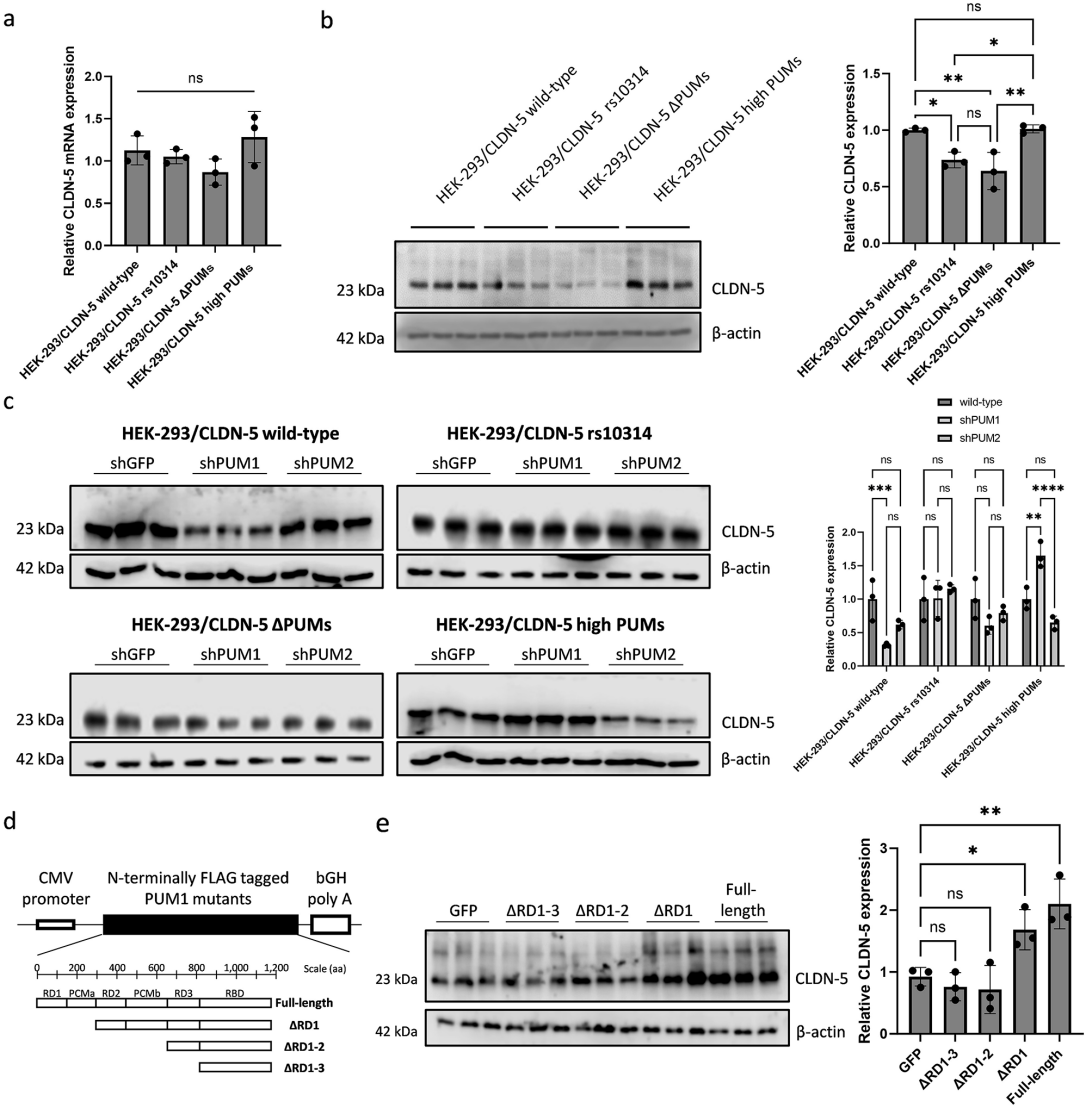
Effect of pumilio proteins on CLDN-5 expression. (**a** and **b**) The expression level of CLDN-5 mRNA **(a)** and CLDN-5 protein **(b)** in HEK-293 cells stably expressing CLDN-5 or its 3’-UTR mutants. Three independent clones were used. **(c)** CLDN-5 expression level was checked 48 h after the transfection of shRNA against GFP, PUM1 or PUM2 into each CLDN-5 expressing HEK-293 cells (*n* = 3). shRNA GFP transfected cells were used as a control. **(d)** FLAG-tagged N-terminally truncated PUM1 mutant expressing vectors. RD; repression domain, PCM; pumilio conserved motif, RBD; RNA-binding domain. **(e)** CLDN-5 expression level in HEK-293 cells stably expressing wild-type CLDN-5 was checked 48 h after the transfection of FLAG-tagged truncated PUM1 mutants expressing vectors. GFP-expressing vector was used for a transfection control. ns, not significant (*p* > 0.05); *p value ≤ 0.05; **p value ≤ 0.01; ***p value ≤ 0.005;****p value ≤ 0.001

To examine the effect of PUM-binding on the expression level of CLDN-5, mRNA and protein levels in four CLDN-5 stably expressing HEK-293 cells were evaluated. mRNA levels were comparable among these four cells (Fig. 2a) consistent with our previous report, however ΔPUM-type CLDN-5 showed slightly lower mRNA levels. Compared to wild-type and high PUM-type CLDN-5 expressing cells, CLDN-5 protein level was less than 75% in rs10314-type and 65% in ΔPUM-type CLDN-5 expressing cells (Fig. 2b). This result clearly suggests that the interaction of PUM around the rs10314 site of CLDN-5 mRNA is important to enhance the translational activity of CLDN-5 mRNA, although, a completely attenuated binding capacity of PUMs in this region may ultimately affect the mRNA levels.

To check whether the translational enhancement of CLDN-5 can be reduced by knockdown of PUMs, shRNA-expressing vectors were transfected into these 4 CLDN-5-expressing cells. The knockdown efficacy of these vectors was up to 70% (Supplementary Fig. S2a and S2b). PUM1 and PUM2 are known to affect the expression of each other through their own PUM-binding sequence in their mRNAs [47] and this phenomenon was clearly observed by shPUM1 treatment in our experiments. In wild-type CLDN-5-expressing cells, shPUM1 could attenuate CLDN-5 expression (Fig. 2c). In high PUM-type CLDN-5 expressing cells, shPUM2 could attenuate CLDN-5 expression strongly. In both rs10314-type and ΔPUM-type, neither shPUM1 nor shPUM2 could attenuate CLDN-5 expression. These results are consistent with data from the RIP assay (Fig. 1c). To confirm the effect of PUM1 on CLDN-5 expression, a vector expressing FLAG-tagged PUM1 or its N-terminally truncated mutants (Fig. 2d) was transfected into wild-type CLDN-5 expressing cells. Interestingly, full-length PUM1 and the repression domain (RD) 1 lacking mutant could enhance CLDN-5 expression while mutants lacking the RD2 and RD3 domains could not (Fig. 2e). To confirm whether these PUMs are functional, granular formation of PUM1 upon oxidative stress induced by sodium arsenite was examined as it is well established that PUMs interact with NORAD (Non-coding RNA activated by DNA damage, an abundant long non-coding RNA with 18 PBEs) to form membrane-less ribonucleoprotein granules, called “stress granules”, upon stress stimuli [12, 28]. All vectors expressing FLAG-tagged PUM1 or its mutants could form granules upon stress stimuli, although PUM1 lacking RD1 to RD3 (ΔRD1-3) showed less efficacy (Supplementary Fig. S3a and S3b). Therefore, the RD2 domain and PUM-conserved motif B (PCMb) are predicted to be responsible for inducing PUM1-mediated CLDN-5 up-regulation. Taken together, these results indicate that PUMs function as a translational enhancer of CLDN-5 but the rs10314-type CLDN-5 mRNA has restricted capacity to gain from this effect.

To check whether these PUMs and 3’-UTR of CLDN-5-mediated translational regulation are also observed in human brain endothelial cells that express endogenous CLDN-5, dual-luciferase assays were performed using hCMEC/D3 cells and vectors that have the 3’-UTR of CLDN-5 downstream of an open reading frame of firefly luciferase (Supplementary Fig. S4a). Similar results were observed by these reporter assays; rs10314 and ΔPUMs mutant attenuated luciferase expression (Supplementary Fig. S4b) and co-transfection of shPUM1 or PUM1 expressing vectors attenuated or enhanced luciferase expression, respectively, from mRNA with the 3’-UTR of wild-type CLDN-5 (Supplementary Fig. S4c and S4d). Granular PUM1 staining upon sodium arsenite treatment could also be observed in hCMEC/D3 cells (Supplementary Fig. S4e). These results show that the PUM1-binding sequence in the 3’-UTR of CLDN-5 can effectively act as an enhancer of *CLDN5* gene expression in human brain endothelial cells.

### CLDN5 mRNA is enriched in PUM-1 positive stress granules under cell stress conditions

**Fig. 3:**
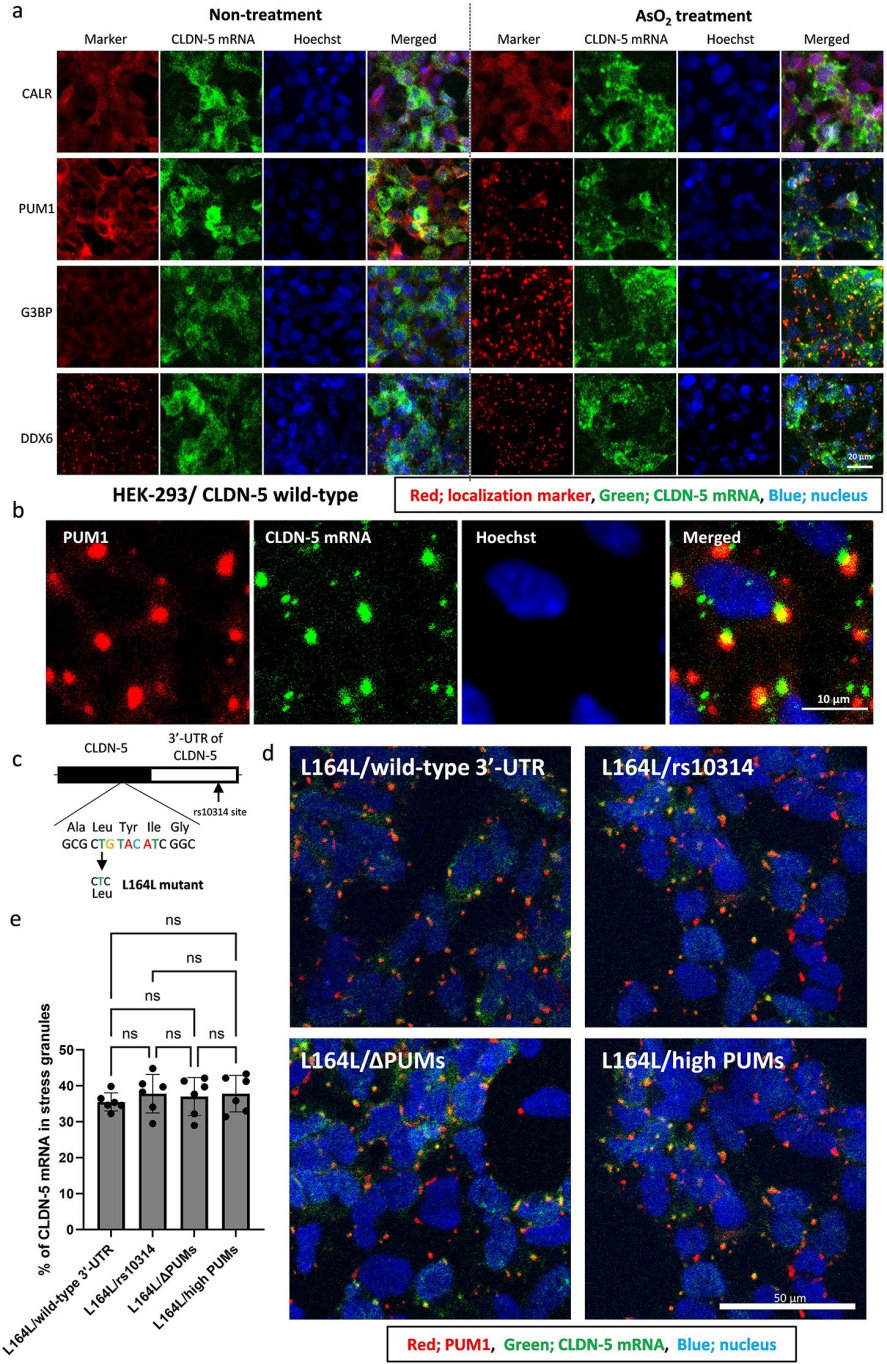
Localization of CLDN-5 mRNA under physiological or cell stress conditions. (**a, b** and **d**) The localization of wild-type CLDN-5 mRNA (**a** and **b**) and its 3’-UTR mutants with L164L mutation **(d)** under the physiological or stressed conditions. To induce the stress granules, the cells were treated with 500 µM of AsO_2_ for 1 h. **(a)** Cells were co-stained with localization marker (red), anti-calreticulin (CALR), anti-pumilio-1 (PUM1), anti-G3BP or anti-DDX6 antibodies. **(b)** A representative higher magnification image of the localization of CLDN-5 mRNA and PUM1 under the stressed condition is shown. **(c)** Information of L164L CLDN-5 mutant. Putative PUM-binding sequence in open reading frame (Leu164 to Ile166) was mutated without an amino acid substitution. The putative PUM-binding sequence in the CDS of CLDN-5 is highlighted by color letters. Consensus PUM-binding element is UGUANAUW (where N is A/U/C/G and W is A/U). **(d)** Cells were co-stained with PUM-1 (red). (**e**) The % of CLDN-5 mRNA in stress granules was calculated in cells expressing L164L CLDN-5 mutants with wild-type 3’-UTR or its mutants under the stressed condition. Nuclei (blue) were counterstained with Hoechst. Data represent means ± SD. ns, not significant (*p* > 0.05)

Stress granules are known to encapsulate 10% of bulk mRNAs, but their encapsulation efficiency is different among mRNA species varying from < 1% to > 95% [28]. To check whether the localization of CLDN-5 mRNA is affected by cell stress conditions, fluorescent in situ hybridization was performed to visualize CLDN-5 mRNA in wild-type CLDN-5 expressing cells. Under physiological condition, CLDN-5 mRNA was distributed within the cytosol and there was no co-localization with DDX6, a marker of P-bodies that is another membrane-less ribonucleoprotein granule (Fig. 3a). PUM1 and G3BP were distributed in the cytosol under physiological conditions. Under cell stress conditions induced by sodium arsenite, CLDN-5 mRNA clearly co-localized with PUM1 and G3BP, a marker of stress granules, and adjacent to DDX6. The cytosolically distributed PUM1 was diminished under cell stress conditions to form the stress granules.

Interestingly, CLDN-5 mRNA has another PBE with one substitution in the CDS where translating ribosomes displace RNA-binding proteins including PUMs [26]. However, the ribosome is halted by global translational suppression under stressed conditions. To precisely assess the effect of the PUM-binding sequence around rs10314 site on mRNA localization under cell stress conditions, a codon was changed without a change of amino acids at L164 to putatively diminish the PUM-binding ability at this site (L164L mutant) (Fig. 3c). However, the L164L mutated mRNA with different 3’-UTRs of CLDN-5 accumulated into stress granules similar to the WT CDS (Fig. 3d); approximately 35% of these CLDN-5 mRNAs were merged with aggregated PUM1 granules (Fig. 3e). These data indicate that CLDN-5 mRNA is accumulated in PUM-positive stress granules under cell stress conditions but it likely occurs in a PUM-binding sequence independent manner. Indeed, many RNA-binding proteins that function as stress granule components such as G3BP1/2, ELAVL1 (HuR), IGF2BP1, TARDBP, and PABPC1 (Youn, Dyakov et al [55]). were highly enriched in RNA-protein pull-down products (Supplementary Table S1).

### Effect of granular formation of PUM1 in human brain tissues

**Fig. 4:**
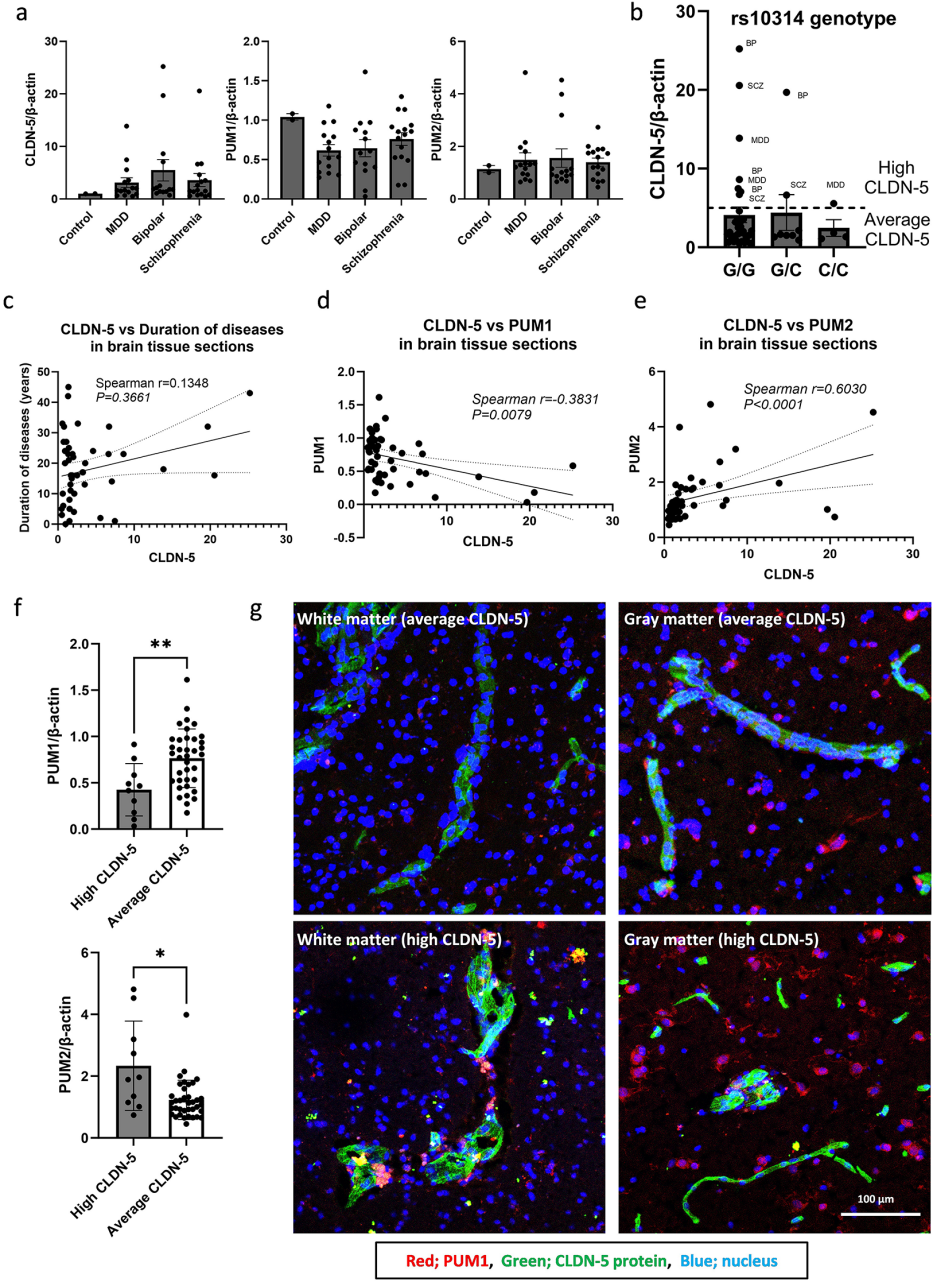
PUM1 granules in post-mortem hippocampal sections of psychiatric patients with high CLDN-5 mRNA levels. **(a** to **f**) The expression level of CLDN-5 mRNA in post-mortem hippocampal sections of psychiatric patients. (**a**) CLDN-5, PUM1 and PUM2 mRNA expression levels among patients. The data represents mean ± SD. (**b**) CLDN-5 mRNA levels were grouped by the difference of rs10314 genotypes. G is wild-type allele and C is rs10314 allele. Patients with higher CLDN-5 mRNA were grouped as High CLDN-5 and their diagnosed psychiatric diseases are indicated in the figure (SCZ; schizophrenia, BP; bipolar, MDD; major depressive disorder). The data represents mean ± SD. The correlation between CLDN-5 mRNA and (**c**) duration of diseases, (**d**) PUM1 or (**e**) PUM2 mRNA levels. Spearman r values and P values are indicated. (**f**) The comparison of PUM1 and PUM2 mRNA levels between high CLDN-5 group and average CLDN-5 group. The data represents mean ± SD. *p value ≤ 0.05; **p value ≤ 0.01. (**g**) Representative confocal images of post-mortem hippocampal sections stained with anti-PUM1 antibody. Red; PUM1, Green; CLDN-5. Blue; nuclei

Previously, we have demonstrated that CLDN-5 protein expression at the BBB was reduced in donor brain tissues from patients with psychiatric disorders [16] and resected brain tissue from patients with temporal lobe epilepsy (Greene, Hanley et al [17]). Based on our accumulated data, we sought to examine whether granular PUM1 can be impacted in the CLDN-5 translational efficacy in patients with psychiatric disorders and epilepsy. Stress granules have a cytoprotective role but persistent formation of stress granules translationally suppresses their components [53]. CLDN-5, PUM1 and PUM2 mRNA levels in the post-mortem hippocampal sections of patients with bipolar (BP), schizophrenia (SCZ) and major depressive disorders (MDD) were measured (Fig. 4a) as BBB integrity in the hippocampus is especially vulnerable to oxidative damage and other stress related stimuli [41]. Interestingly, some patients showed abnormally high CLDN-5 mRNA expression levels, although those patient derived sections showed significantly lower CLDN-5 protein expression level in our previous study [16]. These patients with higher CLDN-5 mRNA level were categorized as a “high CLDN-5 group”. There was no clear association between the high CLDN-5 mRNA levels and rs10314 genotype in the patients (Fig. 4b and Supplementary Table S2). CLDN-5 expression levels were not related with the duration of psychiatric disorders (Fig. 4c), but they were negatively correlated with PUM1 levels (spearman *r* =-0.3831) and positively correlated with PUM2 levels (spearman *r* = 0.6030) (Fig. 4d and e). There were significant differences in PUM1 or PUM2 expression levels between “high CLDN-5” and “average CLDN-5” groups (Fig. 4f). These results indicate that CLDN-5 mRNA in a subset of patients may be translationally suppressed by trapping the stress granules. Therefore, we sought to examine whether granular PUM1 could be observed in the vasculature in these brain sections. As PUM1 is known to be expressed in many cell-types [15]. the immunohistological staining pattern of PUM1 in the brain is poorly understood to date. The cytosolic distribution of PUM1 was mainly observed in the cell-bodies of neurons in the gray matter (Fig. 4g and Supplementary Fig. S5) similar to PUM2 [54]. and to a lesser extent in larger vessels in brain sections. However, the extensive granular staining pattern was observed in larger vessels, but not in capillaries, only in patients with “high CLDN-5” in both white and gray matters. Taken together, these data clearly show that translational suppression and accumulation of CLDN-5 mRNAs by attenuation of PUM1 expression, loss of cytosolic PUM1 and/or PUMs-containing stress granules in larger vessels can be associated in a subset of psychiatric disorders.

**Fig. 5:**
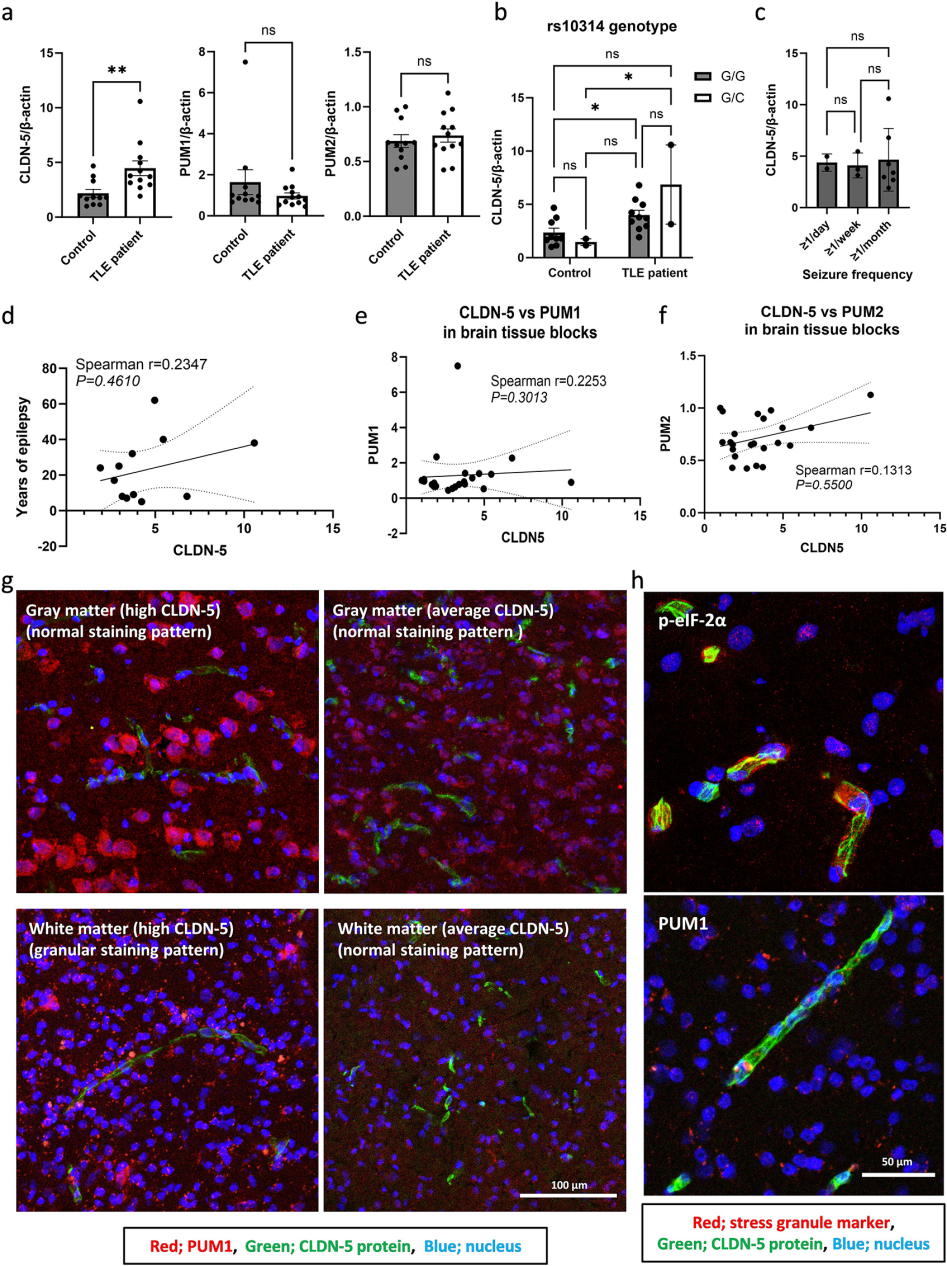
Histological localization of PUM1 in brain tissue from patients with epilepsy. (**a** to **f**) The expression level of CLDN-5 mRNA in resected human brain samples. The RNAs were isolated from the resected brain tissues obtained from control subjects (*n* = 11) and patients with epilepsy (*n* = 12). (**a**) CLDN-5, PUM1 and PUM2 mRNA expression levels between samples collected from control and epilepsy patients. CLDN-5 mRNA levels in the samples were broken down by (**b**) the difference of rs10314 genotypes (G is wild-type allele and C is rs10314 allele) and (**c**) seizure frequency. The data represents mean ± SD. ns, not significant (*p* > 0.05); *p value ≤ 0.05; **p value ≤ 0.01. The correlation between CLDN-5 mRNA and (**d**) years of epilepsy, (**e**) PUM1 and (**f**) PUM2 mRNA levels. Spearman r values and P values are indicated. (**g**) Representative confocal images of human brain sections stained with anti-PUM1 antibody. A part of the hippocampal region showed a granular PUM1 staining pattern. Red; PUM1, Green; CLDN-5. Blue; nuclei. (**h**) The evaluation of the induction of stress granules in vascular endothelial cells in human brain sections (high CLDN-5). Sections were stained with anti-CLDN-5 (green) and stress granule marker, anti-p-eIF-2α or anti-PUM1 (red). Nuclei (blue) were counterstained with Hoechst

To explore the possibility that translational CLDN-5 suppression also occurs in patients with temporal lobe epilepsy (TLE), RNAs were isolated from resected brain tissue from patients undergoing neurosurgical resection of their temporal lobe due to treatment resistant epilepsy. These samples along with non-diseased control brain tissues were examined for mRNA levels of CLDN-5, PUM1 and PUM2 (Fig. 5a). CLDN-5 mRNA levels were significantly higher in the patient samples while both PUM1 and PUM2 mRNA levels were comparable between control and patient samples. Importantly, CLDN-5 protein levels in the sections prepared from these same samples were previously shown to be significantly lower in the patient group [17]. There was no relationship between CLDN-5 mRNA levels and the rs10314 allele, seizure frequency, years of epilepsy, PUM1 or PUM2 mRNA levels (Fig. 5b and f). Although the sample number was small, the two-way ANOVA revealed no interaction between rs10314 genotypes and the presence of epilepsy at the CLDN-5 mRNA level. The cytosolic distribution of PUM1 could not be observed in capillary vessels in these sections either; however, the granular staining pattern of PUM1 could be observed even in capillary vessels in some specific regions where chronic stress might be induced in patients with high CLDN-5 levels (Fig. 5g, Supplementary Fig. S6 and patient information is in Supplementary Table S2). Unlike immunohistological localization of a stress granule marker, the phosphorylated eukaryotic initiation factor-2α (p-eIF-2α), granular staining pattern of PUM1 was not endothelial cell-specific (Fig. 5h). Therefore, persistent PUM1-mediated granules were induced in a cell-type independent manner by epileptic stimuli-mediated chronic stress in a subset of patients, indicating that it may induce a brain-region specific translational CLDN-5 suppression.

## Discussion

Here, we clearly show that rs10314-mediated CLDN-5 down-regulation occurs due to the inability of the variant CLDN-5 mRNA to bind to PUM1. More than 900 mRNAs are controlled by PUMs [5, 42]. and PUM1 functions as a translational enhancer of CLDN-5. PUM1 is also known to be expressed in a range of tissues and cell-types [15]. Although cytosolic ‘free’ PUM1 was not detectable in capillaries in human brain sections by immunohistochemistry, PUM1-based stress granules were observed around pathogenic lesions in hippocampal sections in a cell-type independent manner. These stress granules, formed by concentrated PUM1, could trap CLDN-5 mRNA efficiently to protect CLDN-5 mRNA from degradation, but it caused increased CLDN-5 mRNA level without translation. The increased CLDN-5 mRNA level with less translational activity was also induced by pathological stimuli in cultured cells [29], mouse models of ischemia-reperfusion injury [3] and patients with schizophrenia [36]. However, the preferential accumulation of CLDN-5 mRNA into stress granules is an unexpected result because recent findings suggest that longer and AU-rich mRNAs tend to localize into membrane-less ribonucleoprotein granules including P-bodies [8] and stress granules [28], but CLDN-5 mRNA is very short (less than 1,400 bases) with a very high GC content (67.9%). It is, however, not surprising CLDN-5 mRNA has this exceptional characteristic to efficiently protect the brain after acute stresses. The sequences around rs10314 (UGUACAGC) clearly act as a substrate for PUMs, although it is not the canonical PUM-binding motif (UGUANAUW (where N is A, C, G or U and W is A or T)). However, an in vitro study using RNA with mutated canonical PUM-binding sequences revealed that an energetic coupling occurs to increase the binding affinity to PUM when position 5 to 8 of the PUM-binding sequence is CAGC, but not CACC [26]. Added to this, since PUMs can recognize single-stranded RNAs in a sequence-specific manner, the secondary structure of the 3’-UTR and accessibility of PUMs to their target sequences are also highly important factors for PUM-mediated gene regulation [32].

In the most stable structure of the CLDN-5 3’-UTR, the wild-type sequence around rs10314 site is more single-stranded compared to rs10314, indicating that altering the secondary structure may also affect its binding affinity of PUM1. Around the rs10314 site, PUMs may be accessible but not to miRNA with RNA-induced silencing complexes (RISC). SNPs or polymorphisms in canonical PUM-binding sequences have previously been considered as a disease-associated polymorphisms [19] however, to our knowledge, this is the first report that a common SNP changes PUM-binding affinity of a non-canonical PUM-binding sequence. There may be other SNPs that can change the PUM-binding affinity of non-canonical PUM-binding sequences but it is unknown whether the SNP affects gene expression via PUMs due to a matter of accessibility.

PUMs are known as translational repressors that can shorten the poly(A) tail [13]). However, some recent reports clearly showed there were some genes that can be activated by PUMs directly via a PUM-binding sequence [5, 13, 42, 47]. These PUM-activated genes have the canonical PBE and an additional motif (BYCSWCSC (where B is not A, Y is C or T and S is C or G) [5] which is also present in the 3’-UTR of CLDN-5 (CCCGACGC at 250 bases upstream from rs10314 site). The function of this additional motif is unclear, but an RNA-binding protein that can convert the inhibitory effect of PUMs may bind to this motif. The PUM1 mutants lacking RD2 and/or RD3 domains did not strongly compete with endogenous PUM1, indicating that the RD2 and/or the PCMb domain may function to stabilize the interaction of PUM1 and CLDN-5 mRNA via interaction with other binding partners. The function of these domains is poorly understood but it may interact with the 5’-cap structure [52]. Another possible mechanism of PUM-mediated gene up-regulation is the competition to the miRNA-RISC complex-binding, like HuR [21, 43]. The mechanism of this competition is also unknown but the binding site of PUMs and miRNA do not need to be in close proximity. miRNA-200 and − 466 were recently reported as a miRNA’s that can directly bind near to the rs10314 site in human CLDN-5 mRNA [30] but neither miRNAs are expressed in HEK-293 cells [22].

It is still unclear as to the biological benefit of persistent stress granule formation and the difference of the RNA/protein compositions in the stress granules induced by different stress stimuli [28] however, some drugs that can remove persistent stress granules have been studied to treat neurological diseases [53]. Our results clearly showed that both wild-type and rs10314-type CLDN-5 mRNA were easily trapped in stress granules/aggregated PUMs by a PUM-binding sequence independent manner. Similarly, PUMs and NORAD are dispensable for recruitment of the other into stress granules [35] although they strongly interact with each other [12]. Therefore, cytosolic ‘free’ PUM1 is reduced by the up-regulation of NORAD and/or incorporation into the stress granules. NORAD has been shown to be up-regulated by aging, hypoxia, lipopolysaccharide, and hyperlipidemia in endothelial cells [4, 56] all of which are well-known factors that can increase vascular permeabilities. Therefore, PUM1-mediated CLDN-5 regulation likely has two independent pathways. Cytosolic ‘free’ PUM1 in endothelial cells shows neuroprotective function by being able to enhance and promote the translation of CLDN-5 mRNA. Additionally, CLDN-5 expression is translationally suppressed under cell stress conditions by global translational arrest with a lack of cytosolic ‘free’ PUM1. Although PUMs have many targets and are expressed in many cell-types, *PUM1* haploinsufficiency causes epilepsy and developmental delay [14]. Added to this, some enhancers of PUMs could reduce seizure activities in seizure models of *Drosophila* [33]. The role of PUMs in stress granules is not yet known, but it may preferentially control the expression levels of their substrates during disassembly of the stress granules. We hypothesize that persistent stress granule formation may induce brain-region specific translational suppression of CLDN-5 by both a PUM dependent and independent manner. Removing persistent stress granules and increasing cytosolic ‘free’ PUMs may function as a highly novel vascular stabilizer.

Unfortunately, we cannot study the effect of the rs10314 mutation on CLDN-5 expression in the BBB using mice, as the sequence of the 3’-UTR in mouse *Cldn5* is poorly conserved; the possible corresponding sequence around rs10314 (AUG *UGUA*CAGC UGG) in mouse *Cldn5* is CCC *UGUA*GGUA CCA (underline indicates matched RNA sequences). Added to this, the phenotype of mice with a 25% reduction in CLDN-5 is predicted to be much weaker than that of mice with 50% reduction of CLDN-5 that have previously shown significant memory impairment while also showing a lowered threshold of kainic acid-induced epilepsy [17]. Moreover, there is known to be a pool of tight junction ‘free’ CLDN-5 that may function to dynamically maintain the amount of tight junction-associated, stable CLDN-5 [40, 48] indicating that the contribution of PUM1-mediated up-regulation of CLDN-5 translation related to long term protection of the paracellular barrier in brain endothelial cells in mammals. However, many studies have now shown that rs10314 is a very weak risk factor of schizophrenia [1, 18, 39, 45, 51], indicating that rs10314 can lower the “threshold” to induce psychiatric diseases by reducing tight junction ‘free’ CLDN-5 in an additive manner. Recent studies suggest that CLDN-5 expression in the prefrontal cortex of female mice and humans is more easily lowered by social-defeat stress responses via DNA methylation [11] and the prevalence of schizophrenia in individuals with rs10314 is higher in women [20]. Brain region-specific knockdown of CLDN-5 using adeno-associated virus (AAV) in mice has also clearly shown that increased BBB permeability in the prefrontal cortex sufficed to induce depression-like behaviors with impaired memory [11, 18]. Therefore, the regulation of CLDN-5 mRNA by PUM1 may represent a key process in neurological and neuropsychiatric disease mechanisms. Indeed, methods aimed at controlling PUM1 mediated CLDN-5 levels may also hold therapeutic promise.

## Materials & methods

### Vector construction

The sequences of open reading frame of human CLDN-5 with 3’-UTR of wild-type and rs10314 alleles of CLDN-5 were amplified from previously established vectors [18] and inserted into the HindIII/XbaI site of pcDNA3.1 (+) (Thermo Fisher Scientific). Then, the bGH polyadenylation sequence was removed and NotI cutting site was inserted using XbaI/BbsI digestion. To prepare 3’-UTR mutants that show negligible affinity against PUMs or high affinity against PUMs, site-directed mutagenesis was performed around the rs10314 site. CLDN-5 L164L mutants were also prepared by site-directed mutagenesis. To prepare luciferase reporter vector, wild-type or mutated 3’-UTR of CLDN-5 was inserted into the SacI/MfeI site of pmiRGLO dual-luciferase vector (Promega) to remove bGH polyadenylation sequence. The sequences of open reading frame of human PUM1 were cloned from cDNA prepared from human embryonic kidney cells (HEK-293, ATCC) and inserted into the HindIII/XbaI site of pcDNA3.1 (+) or newly created XhoI site and AgeI site in RNA-binding domain (RBD) of PUM1. pU6 vector (Millipore-Merck) was used to construct a short-hairpin RNA (shRNA)-expressing vector using BamHI/BsrGI site. All primer sequences for constructing these vectors are summarized in Supplementary Table S3.

### Cell culture and transfection

HEK-293 cells were cultured in Dulbecco’s modified Eagle’s medium supplemented with 10% fetal bovine serum (Sigma-Aldrich) in a 5% CO_2_ incubator at 37°C. The immortalized human cerebral microvascular endothelial cell line, hCMEC/D3 cells were cultured in complete EGM-2 endothelial cell growth medium (Lonza). Cells (1 × 10^5^ cells) were seeded into a 12-well culture plate (Corning) in 1 mL of growth medium one day before transfection using FuGENE HD (Promega). Three to four biological replicates were performed for each condition. Forty-eight hours after the transfection of 0.5 µg pDNAs, cells were harvested and 10% of which was subjected to RNA isolation for real-time RT-PCR and the remaining were subjected to protein extraction for Western blotting. Several clones of HEK-293 cells stably expressing CLDN-5 wild-type or its 3’-UTR mutants were prepared by transfection of PvuI (NEB) digested linearized vector. These cells were maintained in media containing 500 µg/mL G-418 (Promega). For shRNA-mediated knockdown, 4 pU6-shRNA-expressing vectors listed in Supplementary Table S3 were prepared and pU6-shRNA coding sequence 4 of shRNA PUM1 and PUM2 were selected for further analysis due to their high knockdown efficacy. Forty-eight hours after transfection of 1 µg pU6-shRNA vectors, cells were harvested and 10% of which was subjected to RNA isolation for real-time RT-PCR and the remaining used for protein extraction for Western blotting. For dual-luciferase assay, hCMEC/D3 cells (1 × 10^4^ cells) were seeded into a 96-well culture plate (Corning) a day before transfection and 0.1 µg pmiRGLO dual-luciferase vector with or without shRNA or PUMs-expressing vectors were transfected. Forty-eight hours after the transfection, dual-luciferase assay was performed using the Dual-Glo Luciferase assay system (Promega) and luminescence was measured using SPARK 10 M microplate reader (TECAN).

### RNA-protein pull-down assay

The template DNA with a T7 promoter was prepared by PCR from pcDNA3.1 vectors coding CLDN-5 wild-type or its 3’-UTR mutants. Biotin-labeled RNA was synthesized by TranscriptAid T7 High Yield Transcription Kit (Thermo Fisher Scientific) in a total reaction volume of 60 µL at 37 °C for 2 h with 2 µg of the template DNA along with 1 mM biotin-14-UTP (Millipore). After DNase I digestion, the biotinylated RNAs were purified using E.Z.N.A. Total RNA kit I. To prepare the RNA-conjugated magnetic beads, 20 pmol of biotinylated RNAs was mixed with 0.2 mg of beads (Dynabeads kilobaseBINDER, Thermo Fisher Scientific) at room temperature for an hour, after that unbound RNA was removed. For RNA-protein pull-down assay, the lysates of parent HEK-293 cells were used. Cells were lysed in 100 µL of cold mild lysis buffer (25 mM Tris-HCl pH 7.4, 150 mM NaCl, 1 mM EDTA, 1% NP-40 and 5% glycerol with 1% Protease Inhibitor Cocktail III and RNase inhibitor) for 15 min on ice. Lysed cells were then centrifuged at 15,000 g for 15 min at 4 °C and protein concentration of their supernatant was determined by BCA assay kit (Thermo Fisher Scientific). Two hundred µL of the reaction mixture (20 mM Tris-HCl, pH 7.5, 50 mM NaCl, 2 mM MgCl_2_, 0.1% Tween-20, 15% glycerol and 50 µL of cell lysates (8 mg/mL)) was incubated with RNAs-conjugated beads for an hour at 4 °C with rotation. The beads were washed five times with wash buffer (20 mM Tris-HCl, pH 7.5, 10 mM NaCl, 0.1% Tween-20). The beads were aliquoted to 4 tubes and directly subjected to SDS-PAGE followed by Western blotting or proteomic analysis.

### Mass spectrometry

The proteins on the RNA-conjugated magnetic beads were separated by SDS-PAGE and gels were excised into 1 mm^3^ cubes for in-gel digestion with trypsin. In short, the gel cubes were dehydrated with acetonitrile (ACN), and then, reduction and alkylation were performed before performing trypsin (Promega) digestion. The digested peptides were extracted from gel cubes and lyophilized. The digested peptides were measured using a nano-liquid chromatography coupled with electrospray ionization tandem mass spectrometry (nano-LC-ESI-MS/MS) platform. The peptides were resuspended in 0.1% formic acid (FA) and 1 µg of peptides (20 µL in 0.1% FA) were injected into nano-LC system (Ultimate 3000 nano UHPLC system, Thermo Fisher Scientific) equipped with a trapping column (PepMap C18, 100 Å, 100 μm ×2 cm, 5 μm) and an analytical column (PepMap C18, 100 Å, 75 μm ×50 cm, 2 μm). The gradient was (A = 0.1% FA in water, B = 0.1% FA in 80% ACN) 2–8% B in 3 minutes, from 8 to 20% B in 40 min, from 20 to 40% B in 26 min, then from 40 to 90% B in 4 min. The nano-LC system was connected with a Q Exactive HF mass spectrometer (Thermo Fisher Scientific, USA) with an ESI nanospray. The full scan was performed between 300 and 1,650 m/z at a resolution of 60,000 at 200 m/z.

### Processing of mass spectrometry data and compilation of dataset related to mRNA-binding protein

All raw MS files were analyzed based on a label-free quantification (LFQ) algorithm and searched against human protein database based on the species of the samples using Maxquant. The parameters were set as follows: the protein modifications were carbamidomethylation (C) (fixed), oxidation (M) (variable); the enzyme specificity was set to trypsin; the precursor ion mass tolerance was set to 10 ppm, and MS/MS tolerance was 0.5 Da. Only protein groups whose Andromeda score was greater than 70 were kept for further analysis. The identified proteins were classified into molecular function, biological process and cellular component according to gene ontology using Panther database (pantherdb.org/). The quantitative MS data of mRNA-binding proteins in HEK-293 cells was downloaded from the supplementary material of the reference [2]. The components of stress granules listed in RNA granule database [55] were highlighted with a cumulative confidence score.

### Real-time RT-PCR

RNA was isolated from cells and resected human hippocampal tissue blocks or sections using an EZNA total RNA kit (Omega Bio-tek) and residual DNA was digested by DNase I on column. cDNA was prepared using High Capacity cDNA Reverse Transcription Kit (Applied Biosystems). Real-time RT-PCR was carried out using a FastStart Universal SYBR Green Master (ROX) master mix (Roche). according to the manufacturer’s instructions. PCR was performed in a Step-One Plus Real-Time PCR instrument (Applied Biosystems). RT-PCR conditions were as follows: 95 °C for 10 min, 37 cycles of 95 °C for 15 s, and 60 °C for 30 s. A melt curve stage was added: 95 °C for 15 s, 60 °C for 1 min, and 95 °C for 15 s. The comparative ΔΔCt method was used to quantify changes in mRNA levels between treatment groups. All primer sequences for real-time RT-PCR are summarized in Supplementary Table S3.

### Western blotting

Cells were lysed in RIPA lysis buffer [50 mM Tris-HCl (pH 7.4), 0.1% SDS, 1% NP-40, 0.25% sodium deoxycholate, 150 nM NaCl, 1 mM EDTA] with phosphatase and protease inhibitors (Millipore-Sigma), incubated on ice for 10 min with gentle shaking, and centrifuged at 12,000 g at 4 °C for 20 min to collect the supernatant. Protein concentrations were determined by using the BCA assay kit. Proteins in lysate (20 µg) or on magnetic beads (50 µg) were separated by SDS polyacrylamide gels. Proteins were transferred to methanol-activated polyvinylidene difluoride (PVDF) membranes and blocked for 1 h at room temperature in 5% non-fat milk and Tris-buffered saline containing 0.05% Tween-20 solution (TBST). Membranes were incubated with primary antibodies listed in Supplementary Table S3 overnight at 4 °C; washed twice for 5 min each in TBST; and incubated with HRP-conjugated goat secondary antibodies for 2 h at room temperature. After four 5 min washes in TBST, protein bands were visualized using enhanced chemiluminescence (Advansta) with an image analyzer (LI-COR C-DiGit scanner). Densitometry was performed using ImageJ with protein bands of interest normalized to the loading control β-actin.

### RNA-immunoprecipitation (RIP)

RIP experiments using anti-PUM1 or PUM2 antibodies were performed in HEK-293 cells stably expressing CLDN-5 wild-type or its 3’-UTR mutants or parent HEK-293 cells. Cells were lysed in 100 µL of cold mild lysis buffer as described above for 15 min on ice. Lysed cells were then centrifuged at 15,000 g for 15 min at 4 °C and protein concentration of their supernatant was determined by BCA assay. To prepare antibodies-coupled magnetic beads, 5 µg of antibody (anti-PUM1, abcam, clone EPR3795; anti-PUM2, abcam, clone EPR3813; Rabbit IgG control, Millipore, 12–370) was mixed with 1.5 mg of beads (Protein A Dynabeads, Thermo Fisher Scientific) at room temperature for an hour, after then unbound antibody was removed. Fifty µL of lysate (5 mg/mL) was added to pre-washed and antibody-coupled beads. Sample and beads were incubated at 4 °C overnight with rotation. The next day, beads were washed three times with cold mild lysis buffer and once with PBS. To release RNAs from beads, Trizol (Thermo Fisher Scientific) was used, and then RNAs were further purified using EZNA Total RNA kit I with DNase I treatment. After cDNA synthesis, qPCR was performed. The abundance of CLDN-5 mRNA in each RIP samples was normalized by that of mRNA *DEK*, which is known as a target of PUM1 and PUM2 [5].

### Fluorescence in situ hybridization and immunocytochemistry

hCMEC/D3 cells or HEK-293 cells stably expressing wild-type CLDN-5 or its mutants were seeded into collagen type I coated (BD biosciences) 8 chamber slide (Sarstedt). HEK-293 cells were cultured for 2 days to reach 60 to 80% confluency and hCMEC/D3 cells were cultured 4 days at 100% confluence. For fluorescence in situ hybridization, cells were fixed and permeabilized using the ViewRNA Cell Plus kit (Thermo Fisher Scientifc). To induce stress granules, cells were treated with 500 µM of sodium arsenite (AsO_2_) for 1 h before cells were fixed. The marker proteins in cells were stained using antibodies listed in S. Table 3 and the CLDN-5 mRNA in cells were stained using a probe set (VX-06, assay ID: VA4-3088601-VCP) (Thermo Fisher Scientific). For immunocytochemistry, cells were fixed with 4% PFA for 15 min and then permeabilized with 0.1% triton-X for 5 min. After the washing, blocking was carried out using 1% BSA-PBS. Then, cells were incubated with rabbit anti-PUM1 antibodies (clone EPR3795, Abcam) and mouse anti-FLAG tag antibodies (clone 1E6, Wako Pure Chemicals) in 1% BSA-PBS at 4 °C overnight. Subsequently, cells were washed twice with PBS and incubated with secondary antibodies listed in Supplementary Table S3 in 1% BSA-PBS for 2 h at room temperature. Hoechst was used to visualize nuclei. Images were taken with a Zeiss LSM 710 confocal laser scanning microscope using 20× or 40× objective lens.

To quantify the mRNA level in the PUM1-containing granules, the images of CLDN-5 mRNA and PUM1 were separately binarized using ImageJ. Manually binarized images of PUM1-containing granules were intersected with respective binarized images of CLDN-5 mRNA using ‘AND’ function of Fiji. The resulting intensity of CLDN-5 mRNA was then used to calculate % of CLDN-5 mRNA in stress granules. Six images were used for each group.

### Immunohistochemistry

All ethical approval for experiments using human resected temporal lobe lesion in this study were obtained from relevant local and national component authorities before the initiation of the studies. Resected brain tissue was obtained from refractory temporal lobe epilepsy patients or autopsy control samples provided by the Stanley Medical Research Institute. The St James’ Hospital ethics committee approved the human studies and written informed consent was obtained from all participants. Post-mortem hippocampal sections of schizophrenia, bipolar disorder, major depression disorder and non-diseased control brains were obtained through the Stanley Medical Research Foundation. Samples were collected, with informed consent from next-of-kin, by participating medical examiners between January 1995 and June 2002. Fresh frozen 14 μm sections from resected tissues were fixed for 10 min in ice-cold methanol and subsequently rinsed briefly in PBS. Sections were blocked with 5% normal goat serum (NGS) containing 0.05% triton-x100 in PBS for 1 h at room temperature. The sections were incubated with mouse anti-CLDN-5 antibodies (clone 4C3C2, Thermo Fisher Scientific) and rabbit anti-PUM1 antibodies (clone EPR3795, Abcam) or rabbit anti- p-eIF-2α antibodies (44-728G, Thermo Fisher Scientific) in 1% NGS overnight at 4 °C. Sections were washed twice in PBS and incubated with secondary fluorescent antibodies listed in Supplementary Table S3 in 1% NGS for 2 h at room temperature. Sections were washed with PBS five times and nuclei were counterstained with Hoechst for 1 min at room temperature before a final wash. Sections were cover-slipped with Aqua Poly/mount (Polysciences) and left to air-dry in the dark before imaging. Images were taken on a Zeiss LSM 710 confocal microscope using 20× objective lens.

### Statistical analyses

GraphPad Prism 9.3.1 (GraphPad Software) was used for statistical analyses. Statistical analysis was performed using Student’s t-test, with significance represented by a P value of ≤ 0.05. For multiple comparisons, one-way ANOVA or two-way ANOVA was used with a Dunnett post-test or with a Sidak’s post-hoc test, respectively, and significance represented by a P value of ≤ 0.05. The correlation between CLDN-5 mRNA level and other factors was assessed using Spearman’s rank correlation analysis. A Spearman r value greater than 0.3 or less than − 0.3 with a P value of ≤ 0.05 is considered as correlated.

### Online supplemental material

Supplementary Table S1 shows all detected proteins in proteomics analysis with some additional information. Supplementary Table S2 shows the information of patients and patient-derived samples that we used for qPCR and immunohistochemistry. Supplementary Table S3 shows the sequence of oligo DNAs and the catalogue number of antibodies used in this study. Supplementary Fig. S1 shows the information of secondary structure of CLDN-5 mRNA. Supplementary Fig. S2 shows the knockdown efficacy of shRNA expressing PUM1 or PUM2. Supplementary Fig. S3 shows the functional activity of N-terminally truncated PUM1 mutants. Supplementary Fig. S4 shows the vector information of luciferase vector with 3’-UTR of CLDN-5 mRNA and the series of assay using a human brain microvascular endothelial cell line. Supplementary Figs. S5 and S6 are other representative images related to Figs. 4 and 5.

### Electronic supplementary material

Below is the link to the electronic supplementary material.

Supplementary Material 1

Supplementary Material 2

Supplementary Material 3

Supplementary Material 4

Supplementary Material 5

Supplementary Material 6

Supplementary Material 7

Supplementary Material 8

Supplementary Material 9

## Data Availability

All data is proved within the manuscript and supplementary information.
