# Supplementary figures and images for: Pumilio-1 mediated translational control of claudin-5 at the blood-brain barrier
Source: Fluids Barriers CNS. 2024 Jun 19;21:52. doi: 10.1186/s12987-024-00553-5 (PMC11188261; doi:10.1186/s12987-024-00553-5)

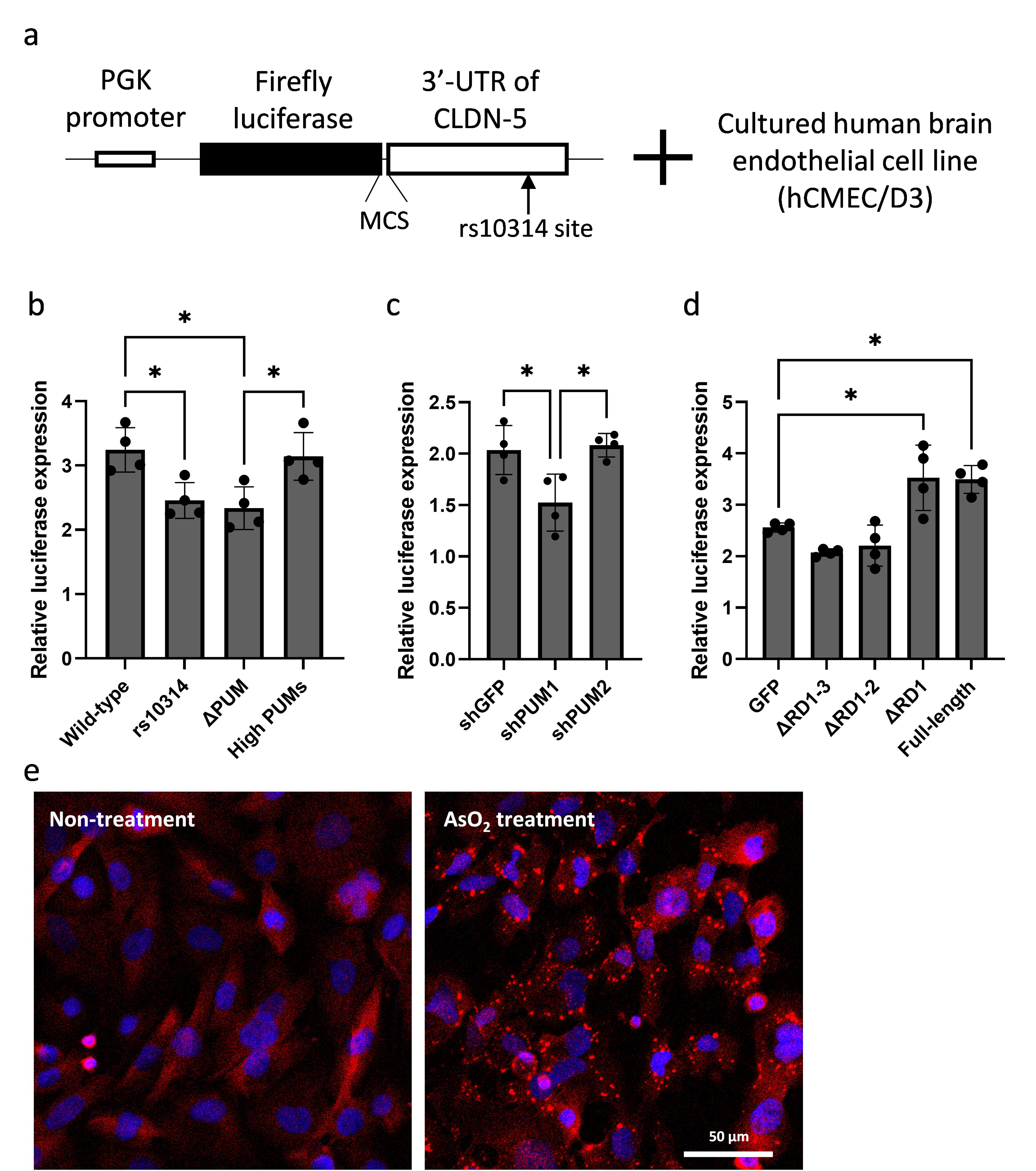

Supplement: Supplementary file 4 — Supplementary Material 4 [file 12987_2024_553_MOESM4_ESM.jpg]

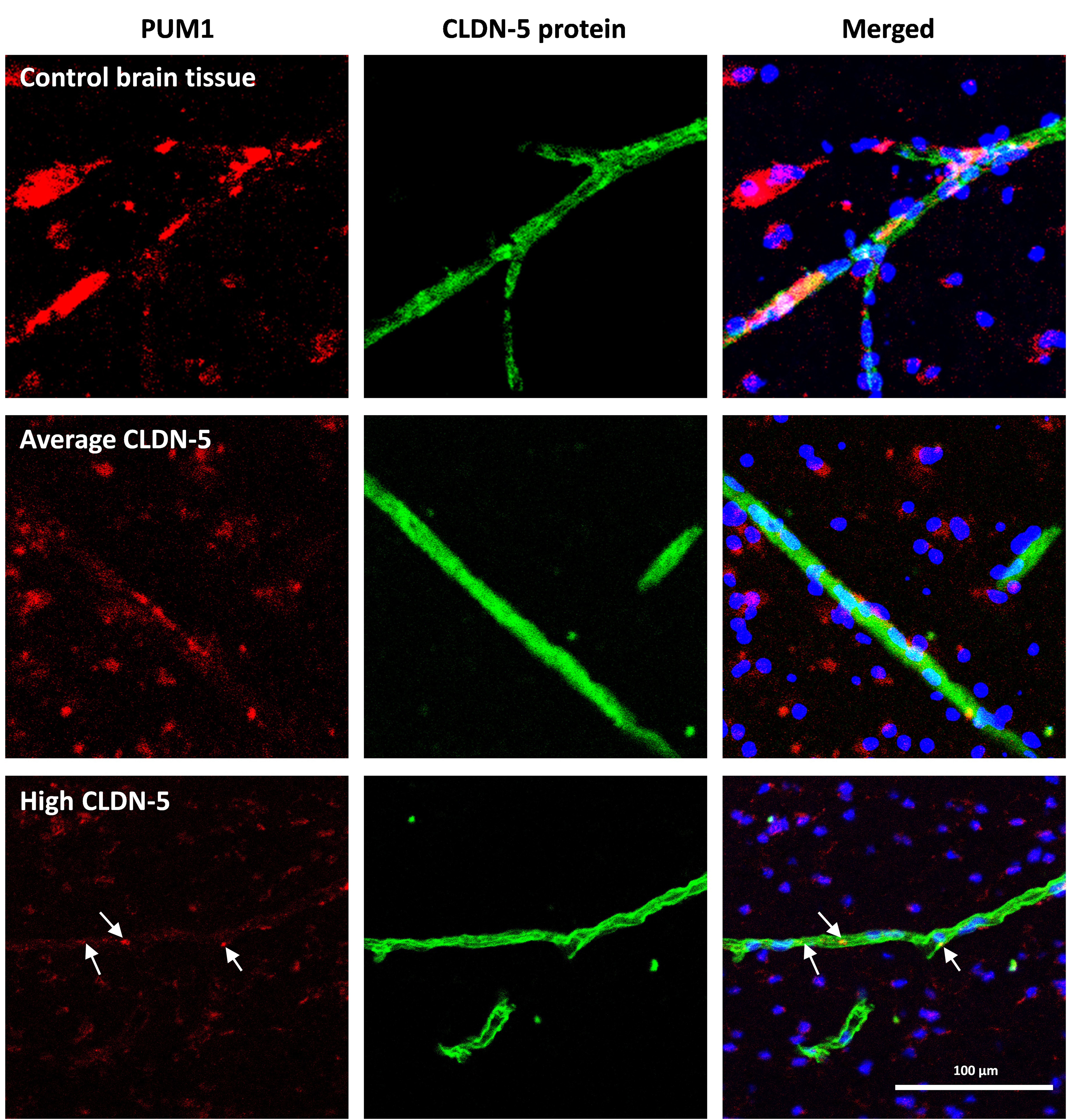

Supplement: Supplementary file 5 — Supplementary Material 5 [file 12987_2024_553_MOESM5_ESM.jpg]

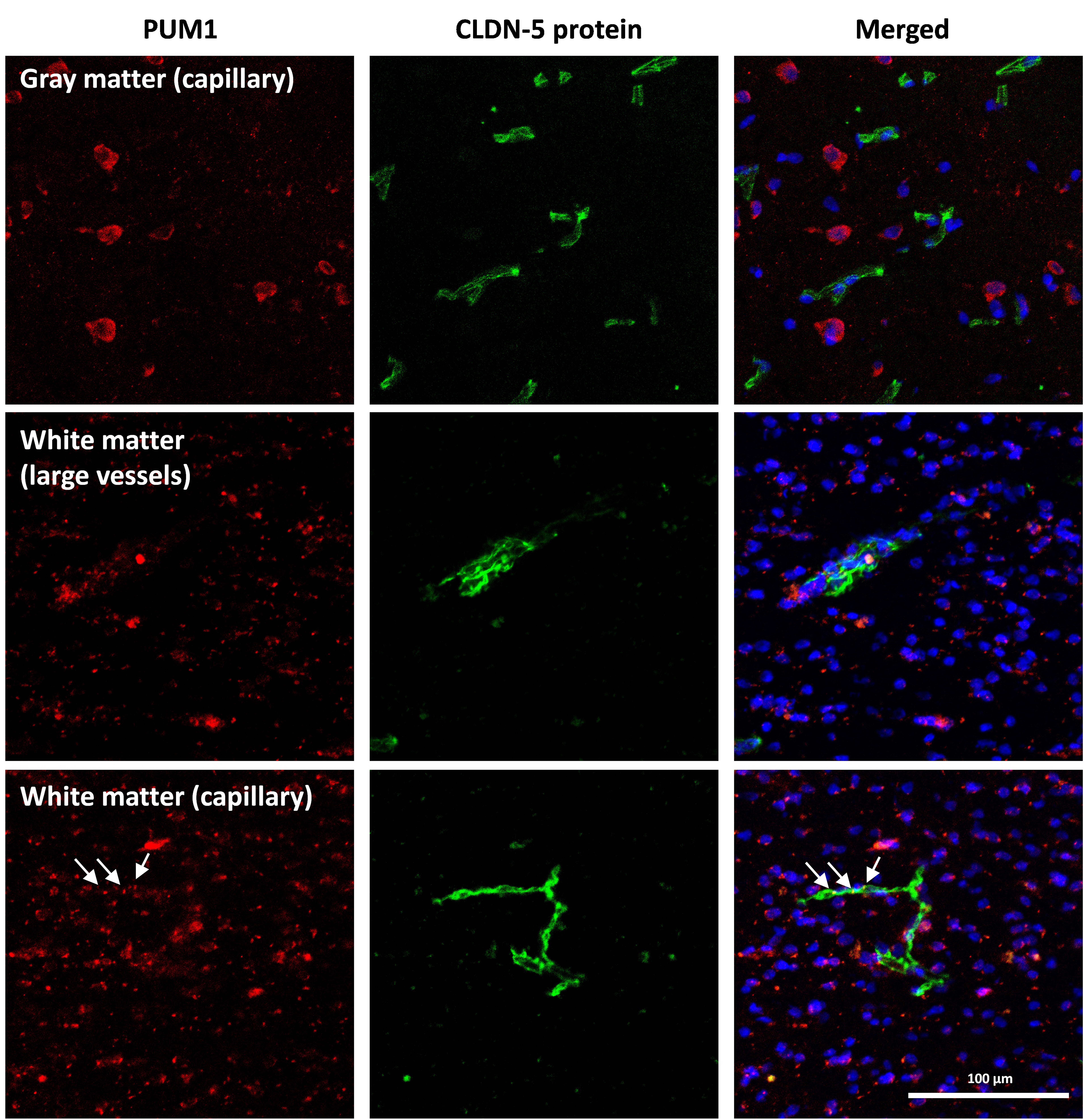

Supplement: Supplementary file 6 — Supplementary Material 6 [file 12987_2024_553_MOESM6_ESM.jpg]
